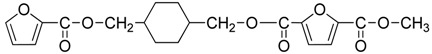# Synthesis and Characterization of Bio-Based Polyesters: Poly(2-methyl-1,3-propylene-2,5-furanoate), Poly(isosorbide-2,5-furanoate), Poly(1,4-cyclohexanedimethylene-2,5-furanoate)

**DOI:** 10.3390/ma10070801

**Published:** 2017-07-14

**Authors:** Zoi Terzopoulou, Nejib Kasmi, Vasilios Tsanaktsis, Nikolaos Doulakas, Dimitrios N. Bikiaris, Dimitris S. Achilias, George Z. Papageorgiou

**Affiliations:** 1 Laboratory of Polymer Chemistry and Technology, Department of Chemistry, Aristotle University of Thessaloniki, Thessaloniki GR-541 24, Greece; terzoe@gmail.com (Z.T.); nejibkasmi@gmail.com (N.K.); vt-vasilis@windowslive.com (V.T.); doulakas@gmail.com (N.D.); axilias@chem.auth.gr (D.S.A.); 2 Department of Chemistry, University of Ioannina, Ioannina GR-54110, Greece

**Keywords:** furanoate polyesters, complicated structure diols, synthesis, thermal properties

## Abstract

In the present study, three new biobased furanoate polyesters with potential use in food packaging applications, named poly(isosorbide furanoate) (PIsF), poly(methyl-propylene furanoate) (PMePF) and poly(1,4-cyclohexane-dimethylene 2,5-furanoate) (PCHDMF) were synthesized. As monomers for the preparation of the polyesters, 2,5-furandicarboxylic acid (FDCA) and diols with irregular or complicated structure were used, including isosorbide (IS), 2-methyl-1,3-propanediol (MPD) and 1,4-cyclohexane-dimethanol (CHDM). The polymerization process was carried out via melt polycondensation method. The structural characteristics and thermal behavior of the polymers were studied. The kinetic fragility of the amorphous phase of the polymers was evaluated. The thermal degradation was studied by means of thermogravimetry and a pyrolysis Py-GC/MS (Pyrolysis-Gas Chromatography/Mass Spectroscopy) system to estimate the degradation mechanism.

## 1. Introduction

The exploitation of fossil based resources and mainly of petroleum to produce petrochemical polymers has as a result the shrinkage of the above. Furfural (F) and 5-hydroxymethylfurfural (HMF) can be isolated from carbohydrates, and they constitute precursor compounds of renewable monomers such as 2,5-diformylfuran (DFF) [1], 5-hydroxymethyl-2-furancarboxylic acid (HMFCA) [2] and FDCA [3]. Poly(ethylene furanoate) (PEF) gains ground, against poly(ethylene terephthalate) (PET), especially in food packaging applications, due to renewable sourcing of the monomers and the largely improved barrier, mechanical, and thermal properties compared to PET [4]. PET is one of the most widely used thermoplastics in the world, especially in bottle and fiber production, and its popularity derives from its versatility, since it can be produced in different molecular weights and easily manipulated crystallinity [5]. Nowadays, there is an increased interest to produce new furanoate polyesters using alternative diols to ethylene glycol [6,7,8]. Besides PEF, poly(propylene furanoate) (PPF) as well as other furanoate polyesters are expected to get their share in the market of food packaging and fibers [7]. Both industry and researchers are showing increased interest in the utilization of furan-based monomers for the replacement of alipharomatic polyesters widely used for decades, such as PET, poly(trimethylene terephthalate) (PTT) and poly(butylene terephthalate) (PBT) [9].

IS constitutes a heterocyclic diol derived from glucose. Due to its complicated structure, rigidity and chirality, it forms amorphous polymers and co-polymers with terephthalate and succinate acid with interesting thermal properties, high *T_g_* values and/or special optical properties [10,11]. PIsF is considered an alternative to polycarbonate (PC), since in contrast to bisphenol A, isosorbide is a non-toxic compound [7]. PC is a widely used thermoplastic with excellent mechanical and thermal properties with applications in automotive industry, data storage, electronics and baby products. By estimation, the production of bisphenol A in 2015 exceeded 5.4 million tons [5].

CHDM is produced by catalytic hydrogenation of dimethyl terephthalate (DMT). The reaction is conducted in two steps beginning with the conversion of DMT to the diester dimethyl 1,4-cyclohexanedicarboxylate (DMCD). In the second step, DMCD is further hydrogenated to CHDM. The cis/trans ratio of CHDM is different depending on the catalyst [12,13]. CHDM is polymerized with terephthalic acid to form a linear polyester, poly(1,4-cyclohexylenedimethylene terephthalate), with improved hydrolytic, chemical, electrical properties, as well as enhanced thermal properties [14]. As a comonomer, CHDM could enhance the mechanical properties, especially toughness, and crystallization of PEF [15].

2-methyl-1,3-propanediol (MPD) is synthesized by isomerization of propylene oxide to allyl alcohol, followed by hydrogenation [16], and is used as an intermediate for organic synthesis, as a solvent and in colorant products.

In the present study, three new furanoate polyesters were synthesized from the reaction of dimethylester of furanoate dicarboxyl acid (DMFD) with MPD, CHDM and IS. For the synthesis of PDMePF and PIsF, a typical two-step procedure was used. On the contrary, an innovate technique was used to achieve the synthesis of PCHDMF due to the higher boiling point of CHDM, as in a previous study of ours [17]. The intrinsic viscosity of the polyesters was measured and the structure confirmation was achieved by Nuclear Magnetic Resonance (NMR) spectroscopy. Thermal properties and thermal stability were determined by Differential Scanning Calorimetry (DSC) and Thermogravimetric Analysis (TGA), respectively, while the existence of degree of crystallinity was investigated by Wide Angle X-ray diffraction patterns (WAXD). Furthermore, the mechanism of thermal degradation during pyrolysis of the three polyesters was studied by Py/GC-MS.

## 2. Experimental

### 2.1. Materials

FDCA (purum 97%), MPD (99%, b.p. = 125 °C), CHDM (mixture of cis-trans, 99%, m.p. = 58 °C and b.p. = 287 °C), Isosorbide (99%, m.p. = 63 °C and b.p. = 160 °C) and Tetrabutyltitanate (TBT) catalyst of analytical grade were purchased from Aldrich Co, Hamburg, Germany.

### 2.2. Polyester Synthesis

PIsF and PMePF were synthesized by the melt polycondensation process, including the transesterification of dimethyl ester of FDCA (DMFD), and its reaction with isosorbide and 2-methyl-1,3-propanediol, respectively, as described in our previous paper [17,18]. Briefly, the monomers were charged into the reaction apparatus with a molar ratio of diester/diol = 1/2 with 400 ppm TBT. The reaction mixture was heated at 150 °C under argon atmosphere for 2 h, at 160 °C for additional 2 h, and finally at 170 °C for 1 h. This first step (transesterification) is considered to be complete after the collection of almost all of the theoretical amount of CH_3_OH, which was removed from the reaction mixture by distillation and collected in a graduated cylinder. After this stage, the corresponding bishydroxyalkylene-2,5-furandicarboxylate monomers have been formed. In the second stage, these monomers reacted with DMFD in a molar ratio 1/1.05 at 150 °C under argon atmosphere for 2 h, at 160 °C for additional 2 h, and finally at 170 °C for 1 h. During this stage, methanol was also removed as byproduct. After that time, in the third step of polycondensation, a vacuum (5.0 Pa) was applied slowly over a period of about 30 min. The temperature was increased to 210 °C, and the polymerization continued for 1 h at this temperature, at 220 °C for 1 h, and at 230 °C for 0.5 h using a stirring speed of 720 rpm. For synthesis of PCHDMF these temperatures were 240, 250 and 260 °C, respectively. After the polycondensation reaction was completed, the polyesters were easily removed, milled, and washed with methanol.

In the case of PCHDMF, a variation of the two-stage melt polycondensation method was applied, due to the high boiling point of 1,4-CHDM, as described in our previous work [17,18].

### 2.3. Polyester Characterization

Intrinsic viscosity [IV] measurements of the prepared polyesters were performed using an Ubbelohde viscometer at 30 °C in a mixture of phenol/1,1,2,2-tetrachloroethane (60/40 *w*/*w*).

^1^H-NMR spectra of polyesters were obtained with a Bruker spectrometer operating at a frequency of 400 MHz for protons using deuteratedtrifluoroacetic acid (d-TFA) as solvent in order to prepare solutions of 5% *w*/*v*. The number of scans was 10 and the sweep width was 6 kHz.

Fourier transform infrared spectroscopy (FTIR) spectra of all the samples were obtained using a Perkin-Elmer FTIR spectrometer, model Spectrum One. The materials were in the form of thin films with thickness of approximately 15 mm. The IR spectra of these films were obtained in absorbance mode and in the spectral region of 400–4000 cm^−1^ using a resolution of 4 cm^−1^ and 64 co-added scans.

WAXD of the samples were recorded using a MiniFlex II XRD system from Rigaku Co., Tokyo, Japan, with CuK*_α_* radiation (*λ* = 0.154 nm) in the angle 2*θ* range from 5 to 60 degrees, after polyester annealing.

For thermal analysis measurements a Perkin–Elmer, Pyris Diamond DSC, coupled with an Intracooler 2P cooling accessory, was used. Samples of 10 ± 0.1 mg sealed in aluminum pans were used, to test the thermal behavior of the polyesters. The samples were heated from −50 °C to 160 °C in a 20 mL/min flow of N_2_ with heating rate 20 °C/min in order to observe the melting temperature of the as received polyesters. The samples first were held at that temperature for 2 min, then quenched and rescanned again till 160 °C. TGA measurements were carried out by a STA 449C (Netzch-Gerätebau, GmbH, Selb, Germany) thermal analyzer from room temperature up to 600 °C with 20 °C/min heating rate and 30 mL/min flow of N_2_ (99.9%).

Thermogravimetric analysis was carried out with a Setsys 16/18 TG-DTA (Setaram Instrumentation, Caluire-et-Cuire, France). Samples (4 ± 0.2 mg) were placed in alumina crucibles and heated from ambient temperature to 650 °C at 20 °C/min in a 50 mL/min flow of N_2_; an empty alumina crucible was used as reference.

For Py-GC/MS analysis of polyesters a very small amount of each material is “dropped” initially into the “Double-Shot” EGA/PY-3030D Pyrolyzer (Frontier Laboratories Ltd., Fukushima, Japan) using a CGS-1050Ex (Frontier Laboratories Ltd, Fukushima, Japan) carrier gas selector. For Evolved Gas Analysis (EGA), the furnace temperature was programmed from 50 to 700 °C with a heating rate of 20 °C/min, using He as purge gas and air as cooling gas. For pyrolysis analysis (flash pyrolysis) each sample was placed into the sample cup which afterwards fell free into the Pyrolyzer furnace. The pre-selected pyrolysis temperature was 600 °C and the GC oven temperature was heated from 70 to 300 °C at 10 °C/min. This temperature was selected based on the EGA. Sample vapors generated in the furnace were split (at a ratio of 1/50), a portion moved to the column at a flow rate of 1 mL/min, pressure 53.6 kPa and the remaining portion exited the system via the vent. The pyrolyzates were separated using temperature programmed capillary column of a Shimadzu QP-2010 Ultra Plus (Shimadzu, Kioto, Japan) gas chromatograph and analyzed by the mass spectrometer MS-QP2010SE of Shimadzu (Shimadzu, Kioto, Japan) use 70 eV. Ultra ALLOY^®^ metal capillary column from Frontier Laboratories LTD (Fukushima Japan) was used containing 5% diphenyl and 95% dimethylpolysiloxane stationary phase, column length 30 m and column ID 0.25 mm. For the mass spectrometer the following conditions were used: Ion source heater 200 °C, interface temperature 320 °C, vacuum 10^−4^–10^0^ Pa, *m*/*z* range 45–500 amu and scan speed 10,000. The chromatograph and spectra retrieved by each experiment are subject to further interpretation through Shimadzu and Frontier post-run software.

## 3. Results and Discussion

### 3.1. Polyester Synthesis

PIsF and PMePF were prepared by applying the two-stage melt polycondensation method (esterification and polycondensation) in a glass batch reactor [18]. The measured [*η*] values are presented in Table 1. The PIsF sample showed [*η*] = 0.39 dL/g, while PMePF showed [*η*] = 0.42 dL/g. On the contrary, to synthesize PCHDMF, a variation of the typical method was applied, as was developed in our previous work [17,18], because 1,4-CHDM is hardly distillable, even after the application of vacuum. Using the traditional method, in the polycondensation stage, temperatures higher than 250–270 °C should be applied prior, to remove the diol byproduct and to increase the molecular weight. However, at such high temperatures, the polyester decomposition would be very extensive, leading to low molecular weight polyesters and coloration, which is also one of the most serious problems in such polyesters. Applying the adjusted procedure, [*η*] = 0.52 dL/g for PCHDMF was achieved. The measured intrinsic viscosities are typical for alipharomatic polyesters and this is proof that the followed procedure can be applied successfully for synthesis of such polyesters.

### 3.2. Structural Characterization

**Figure 1:**
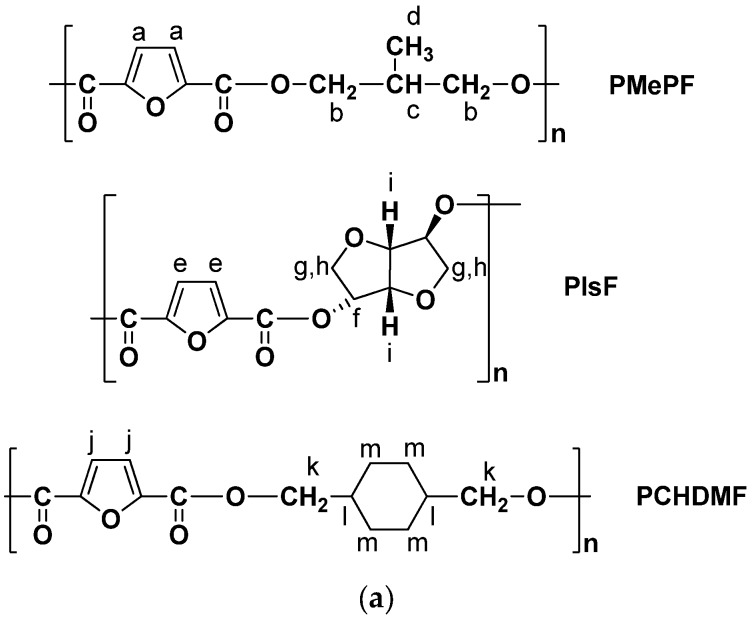

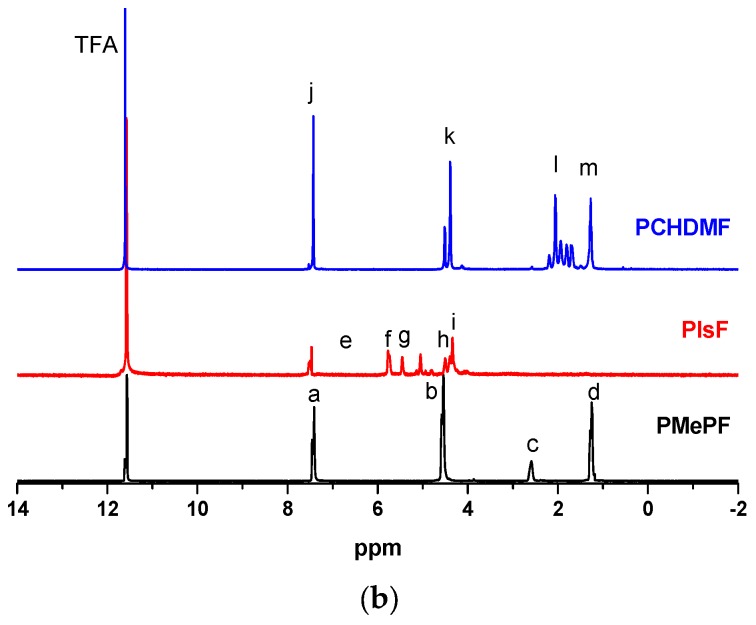
(**a**) Structures; and (**b**) ^1^H-NMR spectra of PMePF, PIsF and PCHDMF polyesters.

The structure of the prepared polyesters was verified with ^1^H-NMR spectroscopy. The spectra of PIsF, PMePF and PCHDMF are shown in Figure 1. At first sight, it is easy to attribute the “a”, “e” and “j” picks to the ring protons at 7.39, 7.46 and 7.44 ppm respectively (2 H, s), due to their highest *π*-deprotection. The PMePF spectra presents a double pick for “b” protons, which are the most deprotected aliphatic protons due to their location next to the ester bond (4 H, d). The “c” proton interacts with 7 protons and gives a multiple peak at 2.58 ppm (1 H, m). The lowest deprotected are the “d” methyl protons and appear at the value of 1.2 ppm (3 H, d).

The isosorbide part of PIsF shows a more complicated spectrum due to its spatial configuration. The most deprotected are the “f” protons owing to their “next to ester bond” location and appear at 5.76 ppm (2 H, q). The *endo* and *exo* “g” protons are more deprotected than the *exo* and *endo* “h” protons, due to the presence of *endo* and *exo* ester bond and appear at 5.76 ppm (2 H, t) and 5.45 ppm (2 H, t), respectively. The “i” protons show a different peak, 4.33 ppm (1 H, d) and 4.50 ppm (1 H, d), as they are characterized as *exo* protons and are in varying degree deprotected by the *endo* and *exo* ester bonds, respectively.

At the PCHDMF spectrum, the “k” protons correspond to two double peaks at 4.38 (2 H, d) and 4.5 ppm (2 H, d), respectively. This phenomenon is attributed to the fact that the used CHMD was a *cis-trans* mixture. Thus, the axial “k” protons at *trans* case interact different than the axial-equatorial “k” protons at *cis* case. The “l” protons show a multiple peak at 2.00 ppm (2H, m) and the “m” protons show a double peak at 1.20 ppm (2 H, d). The absent of any other peaks indicates that polyester with high purity have been prepared.

**Figure 2:**
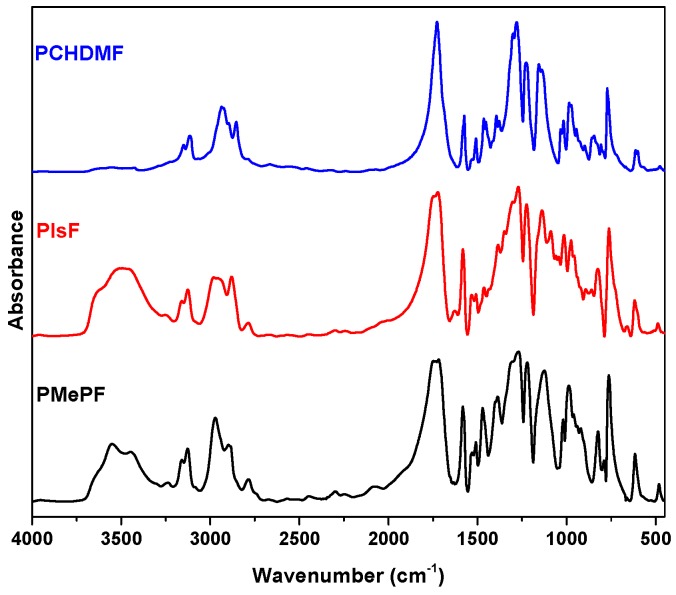
FTIR spectra of the polyesters.

FTIR spectra of the three synthesized polyesters are presented in Figure 2. The spectrum of PMePF shows characteristic peaks at 3550 cm^−1^ due to the stretching of the O–H stretch of the carboxylic end groups, at 3446 cm^−1^ due to the stretching of the hydroxyl end groups, at 2970 and 2900 cm^−1^ due to the C–H stretching, 1730 cm^−1^ due to the C=O stretching of the polyester, 1580 cm^−1^ due to the stretching vibration of the C=C bonds of the furan ring. The asymmetrical bending of the –CH_3_ group of the diol appears at 1470 cm^−1^. The bands of the region 1000–1300 cm^−1^ are caused by stretching and bending vibrations of the C–O bond of the furan ring and the ester moieties. PIsF and PCHDMF present similar absorption bands, with the exception of the –CH_3_ peak of PMePF. PCHDMF exhibits very weak absorption values in the region of 3200–3600 cm^−1^ that corresponds to the –OH and –COOH end groups, which is in agreement with its higher viscosity value reported in Table 1.

**Figure 3:**
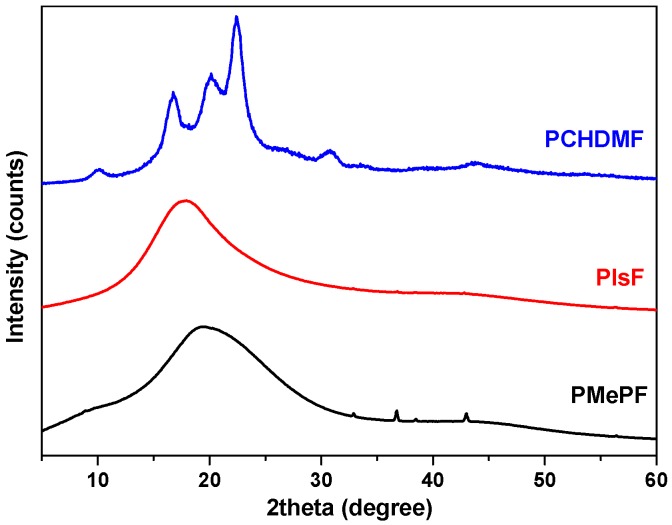
WAXD diffractograms of PMePF, PIsF and PCHDMeF.

The crystalline structure of the polyesters was studied by WAXD. After carrying out a purification process (dissolution in TFA and precipitation by MeOH) followed by annealing (24 h at 100 °C), to achieve the maximum percent crystallinity, only PCHDMF showed crystalline peaks, confessing its semi-crystalline character. Thus, PIsF did not manage to acquire crystallinity, due to *endo* and *exo* hydroxyl groups of isosorbide, which prevent the spatial molecule arrangement. Regarding PMePF, the spherulites formation is rather impossible, due to the methyl group of MPD, which disrupts the molecule symmetry and causes steric hindrance. The WAXD diffraction patterns of the polyesters are presented in Figure 3 and it is observed that the PCHDMF pattern includes some crystalline peaks at the 10–35° range. Specifically, PCHDMF shows peaks at 2*θ* = 10.03, 16.7, 20.19, 22.39 and 30.81°. It seems that there are some similarities in the patterns for PCHDMF and PCHDMT [19].

### 3.3. Thermal Characterization

**Figure 4:**
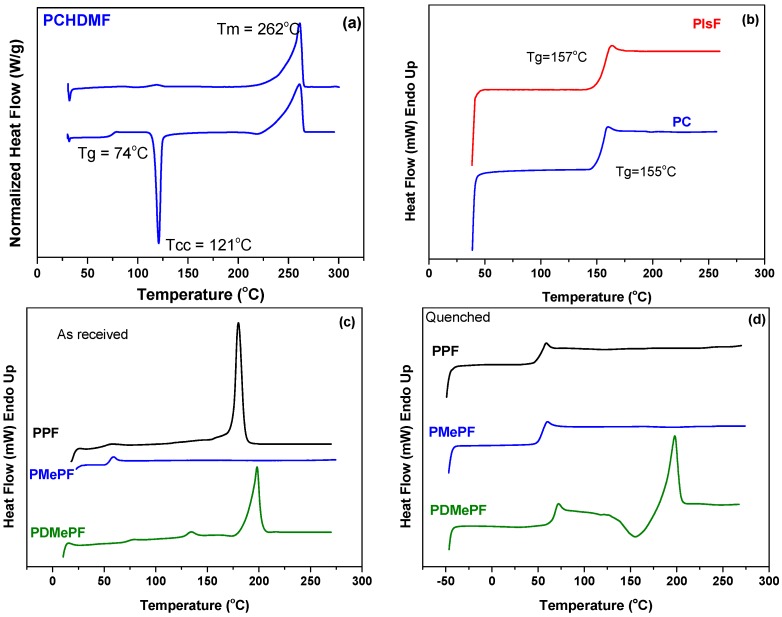
DSC scans of the prepared furanoate polyesters: (**a**) for as received and melt quenched PCHDMF; (**b**) for PIsF and PC; and (**c**,**d**) for as received and melt quenched PPF, PMF, and PDMePF.

The DSC study (Figure 4) revealed the fact that the PCHDMF is a semi-crystalline material, while PIsF and PMePF are amorphous, as was already found by WAXD (Figure 3). PCHDMF showed a high melting temperature (*T_m_* = 262 °C) measured from the first heating scan (Figure 4a). This value is quite higher than that for poly(ethylene furanoate) (PEF) (*T_m_* = 220 °C), but lower compared to that for poly(cyclohexane dimethylene terephthalate) (PCHDMT) [18]. In the DSC trace of the quenched sample a *T_g_* = 74 °C was found. A sharp cold crystallization peak appeared at 121 °C, showing that PCHDMF is a fast crystallizing polymer. This behavior is quite different to those of the most important and most studied furanoate polyesters, poly(ethylene furanoate) (PEF) and poly(propylene furanoate), or even poly(butylene furanoate) (PBF), which crystallize slowly. Only those furanoates from linear aliphatic diols with even number of methylene groups, exceeding six, show fast crystallization, however, their melting temperatures and *T_g_*s are much lower compared to PCHDMF [20,21]. PIsF shows a *T_g_* = 157 °C, which is lower than that reported in a previous work [22], that was synthesized at lower temperature, using dichlorfurandicarboxylate as monomer in a water/DCM system, achieving higher *M_n_*. However, PIsF is considered an alternative to polycarbonate (PC). This *T_g_* value is comparable and even higher than that of PC (Figure 4b). The thermogram of as received PMePF sample revealed only a glass transition, in contrast to PPF and poly(dimethyl propylene furanoate) (PDMEPF) which are crystallizable and showed well defined melting peaks [23]. For PMePF a *T_g_* = 55 °C was evidenced, which is slightly higher than that of PPF, but 13 °C lower in comparison to PDMEPF. The addition of substituents methyl groups, gradually decreases the mobility to the macromolecular chains.

### 3.4. Prediction of Dynamic Fragility through Isoconversional and Standard Methods

#### 3.4.1. Variation of the Effective Activation Energy throughout the Glass Transition Temperature

The variation of the effective activation energy (Δ*E_X_*) through the glass transition can be determined as a function of the extent of conversion using isoconversional methods. According to the Flynn-Wall-Ozawa (FWO) method [24,25], the value of Δ*E_X_* at each value of conversion is calculated as shown in the following equation.

(1) $${\left(\frac{\Delta {Ε}_{x}}{R}\right)}_{x}=-{\left(\frac{d\left(\ln\left(\beta \right)\right)}{d\left(1/T\right)}\right)}_{x}$$

where *β* is the cooling rate, *R* is the universal gas constant and *T* the temperature where the conversion *X* is attained.

Δ*E_X_* can be measured upon cooling through the glass transition using the above equation as thermodynamic properties such as *C_p_* exhibit a monotonic decrease upon transformation from an equilibrium liquid to a glass; however, when measured on heating, *C_p_* often shows an overshoot near the glass transition and the size of the enthalpic overshoot is influenced by thermal history and the heating rate [26]. To estimate the effective activation energy through the glass transition region using an isoconversional analysis, the conversion, *X*, has to be defined. The extent of conversion, *X*, needed for the computations is determined as the normalized heat capacity, $C_{p}^{N}$, that is determined from DSC scans as

(2) $$C_{p}^{N}=\frac{{\left(C_{p}-C_{pg}\right)}_{T}}{{\left(C_{pe}-C_{pg}\right)}_{T}}\equiv X$$

where *C_pg_* and *C_pe_* refer to the glassy and the equilibrium (liquid) heat capacities, respectively. *T_g_* is calculated from DSC measurements as the temperature where the step change in $C_{p}^{N}$ attains half the value of the total change.

Note that this definition is the same with that used by Vyazovkin et al. [10,27] while opposite to that used by Badrianarayanan et al. [26,28] for evaluating cooling curves where *X* = 0 is taken to be the liquid and *X* = 1 is taken to be the glass.

**Figure 5:**
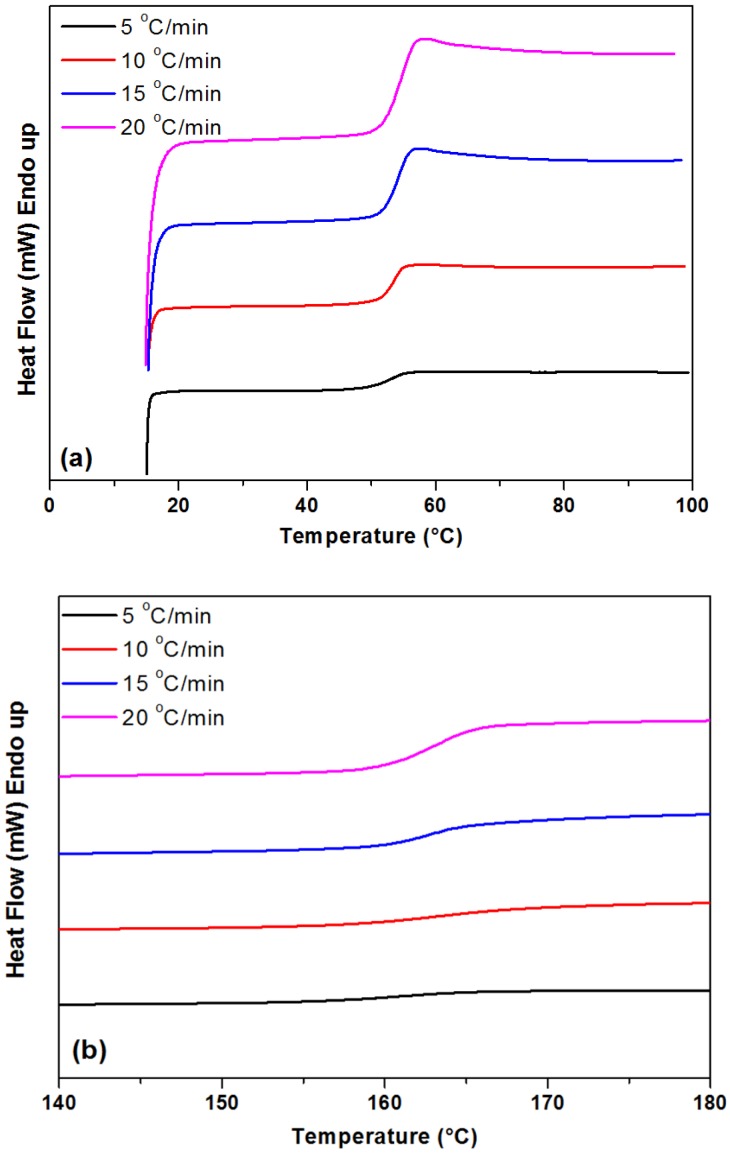
DSC scans of: PMEPF (**a**); and PIF (**b**) glass transition temperature at various heating rates.

**Figure 6:**
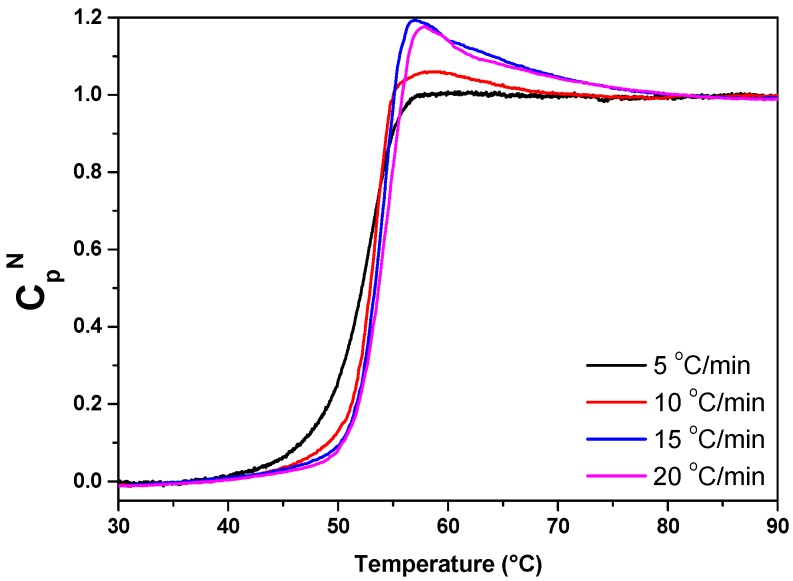
Normalized heat capacity vs. temperature for PMEPF at various heating rates.

Figure 5 displays DSC data taken in the vicinity of *T_g_* at different heating rates. By transforming DSC data to the normalized heat capacity, $C_{p}^{N}$ vs. *T* data sets similar to that shown in Figure 6 are obtained.

**Figure 7:**
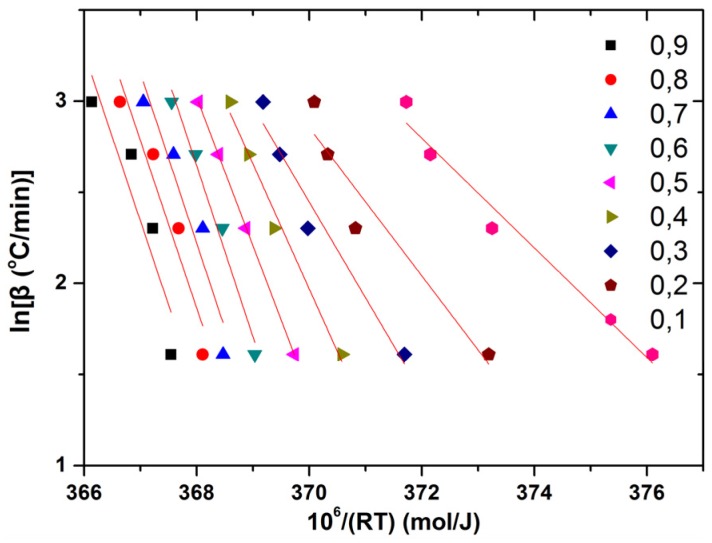
Typical plots for the estimation of the effective activation energy at different relative conversion values.

**Figure 8:**
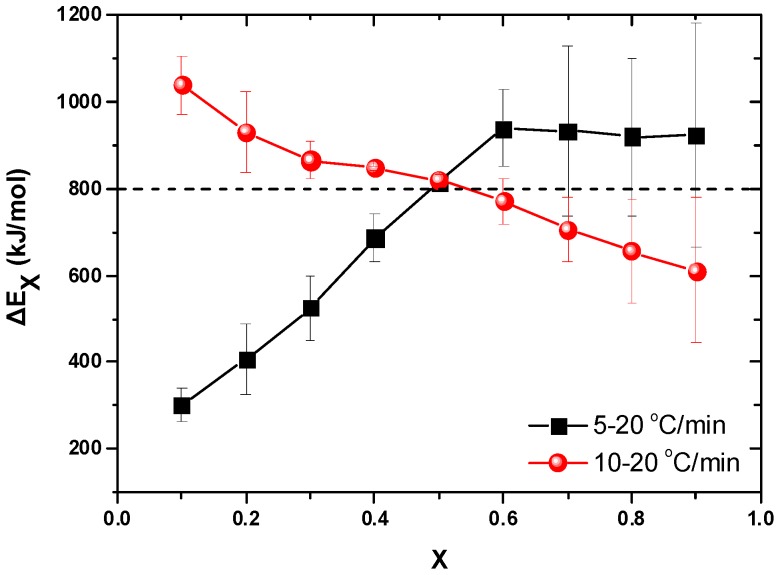
Variation of the effective activation energy with conversion at two different heating rate regimes (i.e., 5–20 and 10–20 °C/min).

Application of the FWO isoconversional method to the $C_{p}^{N}$ vs. *T* data yields the effective activation energy at different degrees of *X*. Typical plots of ln(*β*) vs. 1/*T* at different *X*s appear in Figure 7. The estimated values of ΔEx using two different heating rate regimes are plotted as a function of *X* in Figure 8. When employing the full heating range the values increased with *X*, while when using 10–20 °C/min the dependency demonstrated a decrease with the extent of conversion from the glassy to the liquid state that is in agreement with literature findings [29].

**Figure 9:**
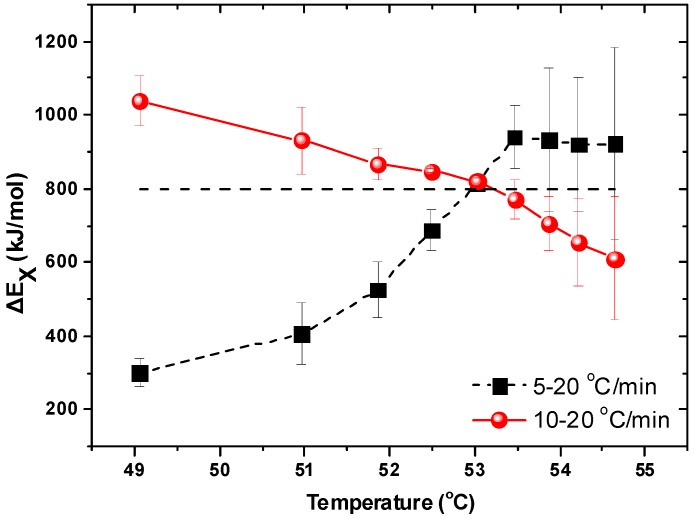
Variation of the effective activation energy with temperature at two different heating rate regimes (i.e., 5–20 and 10–20 °C/min).

The Δ*E_X_* dependencies can be further converted to the dependencies of Δ*E_X_* on *T*. This is accomplished by replacing *X* with an average of the temperatures corresponding to this *X* at different heating rates [10,27]. The resulting temperature dependencies are presented in Figure 9.

Vyazovkin et al. [27], to correlate the effective activation energy with the dynamic fragility of a sample, introduced a variability parameter, Δ*_E_*, denoting the rate of change of Δ*E_X_* with temperature and defined as

(3) $$\Delta_{Ε}=\frac{\Delta {Ε}_{0.25}-\Delta {Ε}_{0.75}}{{Τ}_{0.25}-{Τ}_{0.75}}$$

where Δ*E*_0.25_ and Δ*E*_0.75_ are the effective activation energy values at *X* = 0.25 and 0.75, respectively; and *T*_0.25_ and *T*_0.75_ are the values of *T_X_* for the respective values of *X*. Then, from the values reported in Figure 9, the variability parameter for both polymers can be estimated, as reported in Table 2.

Moreover, other than isoconversional methods expect the value of the effective activation energy to remain constant throughput the glass transition. In order to determine thus an average effective activation energy Δ*E_ave_*, Moynihan et al. [29] have proposed the use of a dependence of the *T_g_* on the rate of heating or cooling in accord with Equation (1) as

(4) $$\frac{\Delta {Ε}_{ave}}{R}=\frac{d\left(ln\left|\beta \right|\right)}{d\left(1/T_{g}\right)}$$

For heating, Equation (4) is applicable subject to the constraint that, prior to heating, the glassy material should be cooled from above to well below the glass transition region at a rate whose absolute value is equal to the rate of heating.

**Figure 10:**
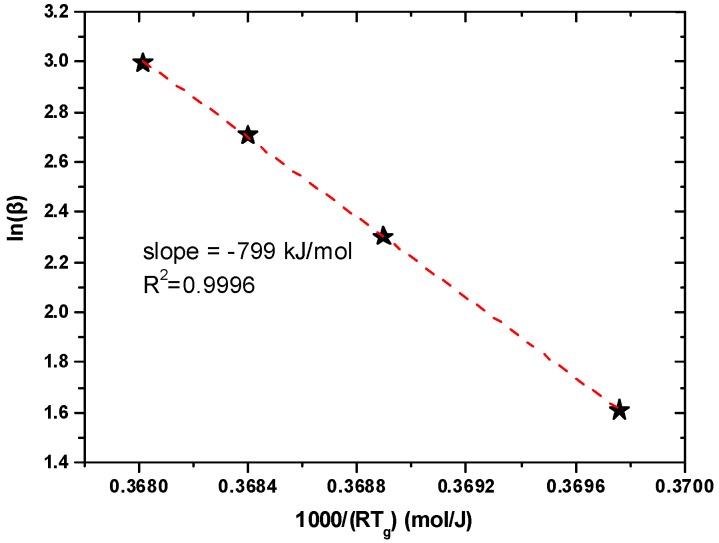
Estimation of the average activation energy at the glass transition for PMePF.

In Figure 6, the glass transition temperatures were measured at different heating rates and, using Equation (4), the average effective activation energy was estimated using either the 5 to 20 °C/min heating rate rang or the 10 to 20 °C/min (Figure 10). The resultant values were similar, and are plotted as straight lines in Figure 4 and Figure 5. These follow the corresponding values estimated from the isoconversional methods.

#### 3.4.2. Prediction of the Dynamic Fragility

The dynamic fragility of PMEPF and PIF were estimated using the scanning rate dependency of *T_g_* method, reviewed in the paper of Crowley and Zografi [30].

Accordingly, at a single temperature, the fragility parameter, *m*, could be defined by [30]:

(5) $$m\equiv {\frac{d\log\tau }{d\left(\frac{T_{g}}{T}\right)}|}_{T=T_{g}}=\frac{\Delta {Ε}_{T_{g}}}{(\ln10)RT_{g}}$$

where *τ* is a mean relaxation time given by the following form of the Vogel–Tammann–Fulcher (VTF) equation:

(6) $$\tau =\tau_{0}\exp\left(\frac{DT_{0}}{T-T_{0}}\right)$$

*τ*_0_, *D* and *T*_0_ are constants, with *D* termed the strength parameter, with a large value (>30) representing “strong” behavior and low *D* value (<10) representing “fragile” behavior. According to the assumptions made in Ref. [30], parameter *D* can be calculated by Equation (7) using *m*_min_ = 16.

(7) $$D=\frac{(\ln10)m_{min}^{2}}{m-m_{min}}$$

In Equation (5), Δ*E_Tg_* is equal to the average effective activation energy, Δ*E_ave_* calculated from the variation of *T_g_* with the heating rate, Equation (4). Therefore, using the value estimated in the previous section for Δ*E_Tg_*, *m* is calculated from Equation (5) and D from Equation (7). All these values appear in Table 1. A large *m* value indicates rapidly changing dynamics at *T_g_* which equates to “fragile” behavior. Therefore, it seems that PMEPF is more fragile than PIF and both are much more fragile compared to PEN. However, PMePF seems to have a behavior similar to PET, with the latter being slightly more fragile. The average effective activation energy for both PMePF and PIF are similar though the large different in the average *T_g_* results in a different behavior concerning fragility. According to the D values, all polymers are considered fragile.

### 3.5. Thermal Degradation

**Figure 11:**
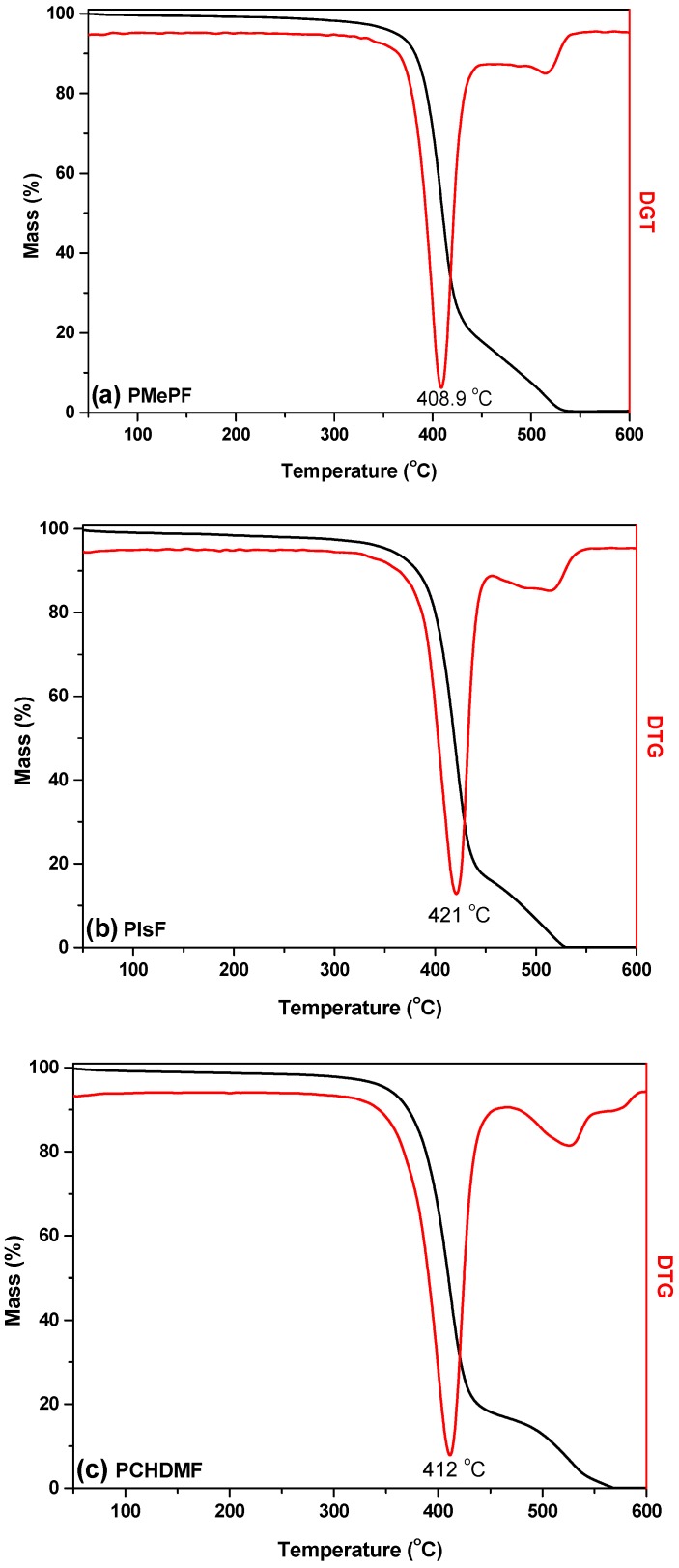
Remaining mass (TG) and differential (dTG) curves of (**a**) PMePF, (**b**) PIsF and (**c**) PCHDMF.

Thermal stability of the polyesters was studied by means of TGA, and the respective thermogravimetric (TG) and differential TG (dTG) curves are presented in Figure 11. As can be seen, all polyesters decompose in two steps, while the maximum rate of decomposition (stage of carbonization) appears at T*_d_*_.*max*_ = 408.9 °C, 421.9 °C and 412.00 °C for PMePF, PIsF and PCHDMF, respectively, while a secondary mass loss takes place at 516.63 °C, 518.49 °C and 526.75 °C respectively, as follows from dTG. It is clear that all polymers are thermally stable materials, which was also proven for other furan polyesters in our previous studies [31,32].

### 3.6. Decomposition Mechanism Study by Py/GC-MS

**Figure 12:**
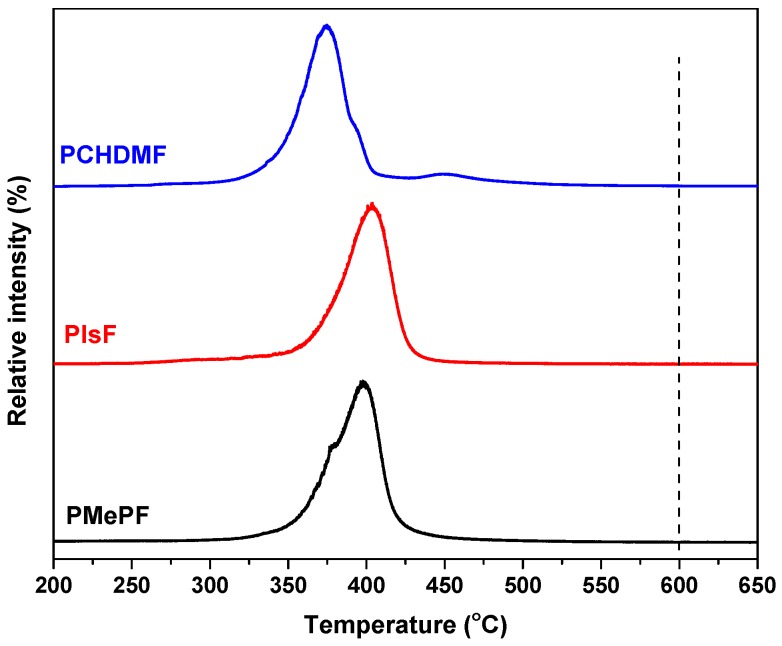
EGA analysis of the polyesters.

**Figure 13:**
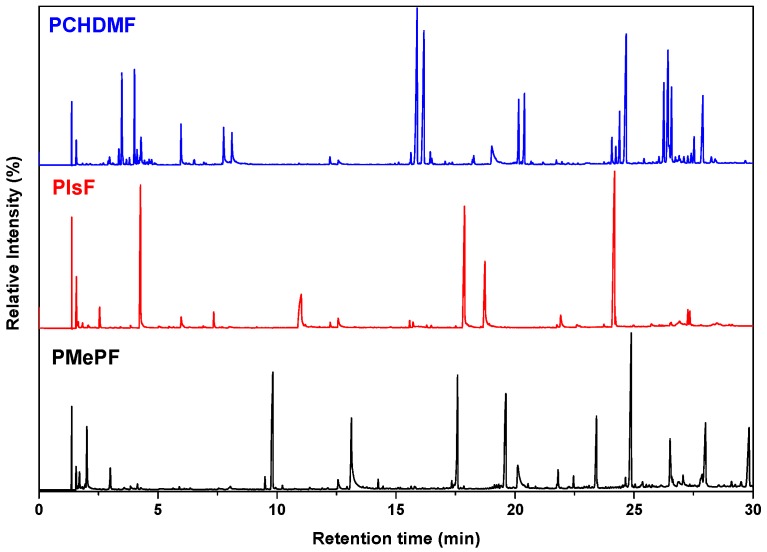
Gas chromatographs of the polyesters after pyrolysis at 600 °C.

To study the decomposition mechanism of the prepared polyesters in detail, characterization with Py/GC-MS was employed. Initially, EGA was performed and the flash pyrolysis temperature was selected based on the resulting thermograms of Figure 12. The evolution of gases for the polyester starts above 325 °C, and ends after 500 °C. Therefore, the selected temperature for flash pyrolysis was 600 °C, where all polyesters were fully decomposed. The resulting chromatographs, after pyrolysis at 600 °C are presented in Figure 13. Each peak corresponds to a mass spectrum that corresponds to a decomposition product, and the most important identified compounds for each polyester are presented in Table 3, Table 4 and Table 5.

Understanding the decomposition mechanism of polyesters is of great importance since it can help with choosing the most suitable methods or fillers to enhance their thermal stability. Pyrolysis of several furanic polyesters has been widely studied by our research team [23,31,33,34], while the case of PMePF, PIsF and PCHDMF is reported for the first time in this study.

In general, furanic polyesters degrade similarly to terephthalate polyesters, with *β*-hydrogen heterolytic scission being the dominant mechanism when *β*-hydrogens are present in the structure of the macromolecular chains. Secondary mechanisms that occur mostly at elevated temperatures are *α*-scission reactions and homolytic processes.

**Scheme 1:**
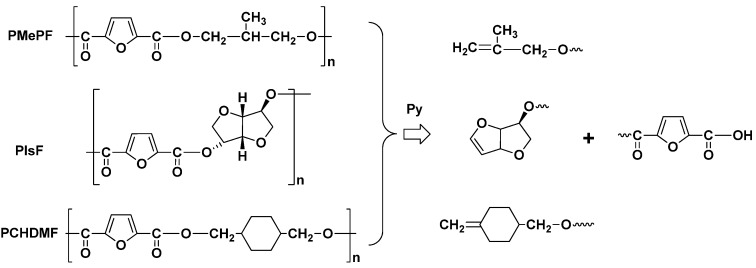
Heterolytic *β*-hydrogen scission degradation mechanism.

In small retention times (Rt), volatile molecules of low molecular weights (MW), such as carbon dioxide, furan and 2-furoic acid were identified. As the Rt increases, bigger and more complex structures, such as dimers, were identified. The major products correspond to compounds with vinyl- and carboxyl- end groups. Those groups are characteristic products of *β*-scission reactions, presented in Scheme 1. Additionally, the used monomers (DMFD or MFD, DMP, Is, CHDM) were detected. Compounds with methyl-ester end groups resulted from the esterification step of the synthesis procedure. Regarding the chromatograph pattern of PCHDM, the appearance of double peaks is noteworthy. Those correspond to cis-trans isomers, since the used diol CHDM is a mixture of the two different stereoisomers.

## 4. Conclusions

Poly(isosorbide furanoate) (PIs), poly(methyl-propylene furanoate) (PMePF) and poly(cyclohanedimethylene furanoate) (PCHDMF) were synthesized using the melt polycondensation method and a variation of it, as verified by ^1^H-NMR spectroscopy and IV measurements. PIsF and PMEPF polyesters are amorphous and showed *T_g_* at 157 °C and 55 °C, respectively. PCHDMF is semi-crystalline material with *T_m_* = 262 °C and *T_g_* = 74 °C. All three polyesters exhibit high thermal stability, since they degrade at temperatures higher than 300 °C. The degradation mechanism was explored. Kinetic fragility was calculated for the amorphous materials.

## Figures and Tables

**Table 1:** Intrinsic viscosity values of the polyesters.

Polyester	[*η*] (dL/g)
PMePF	0.42
PIsF	0.39
PCHDMF	0.52

**Table 2:** Fragility parameters for different polymers.

Material	Δ*E_Tg_* (kJ/mol)	*T_g_* (°C)	m	D	Δ*_E_* (kJ/mol/K)
PMePF	799 ± 14	53.0	128	5.26	−82.6
PIsF	810 ± 46	161.9	97.2	7.26	−32.4
PET [10]			156		−110.6
PEN [29]	451	123	60	13.5	−15.6

**Table 3:** Identified pyrolysis products of PMePF.

Rt (min)	MW (amu)	Possible Structure
1.37	44	CO_2_
1.56	68	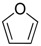
1.70	70	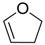
2.01	90	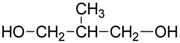
3.00	112	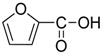
9.49	168	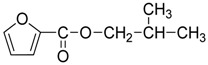
9.82	166	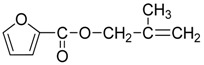
12.56	162	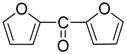
13.13	184	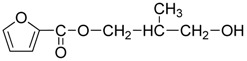
17.59	264	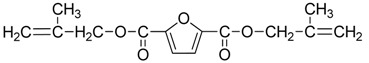
19.62	278	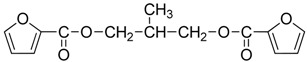
23.43	322	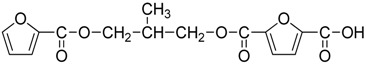
24.64	378	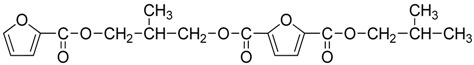
24.88	376	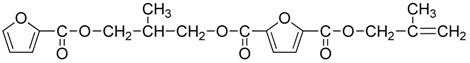
26.52	389	unidentified
28.01	420	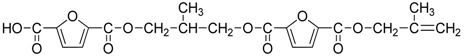
29.84	474	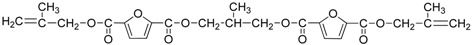

**Table 4:** Identified pyrolysis products of PIsF.

Rt (min)	MW (amu)	Possible Structure
1.38	44	CO_2_
1.57	68	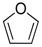
4.26	112	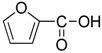
5.97	126	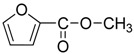
7.34	128	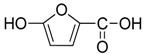
11.02	146	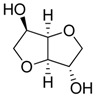
12.57	162	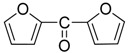
17.88	180	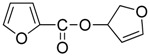
18.74	197	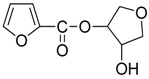
24.19	222	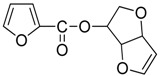

**Table 5:** Identified pyrolysis products of PCHDMF.

Rt (min)	MW (amu)	Possible Structure
1.37	44	CO_2_
1.56	68	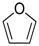
3.48	108	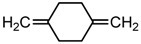
4.01	108	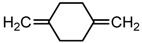
5.97	127	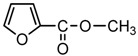
7.76	144	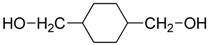
8.11	144	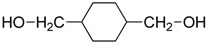
15.89	192	unidentified
16.18	112	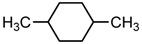
20.16	250	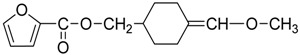
20.40	247	unidentified
24.24	296	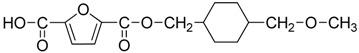
24.40	332	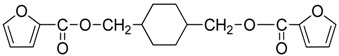
24.67	332	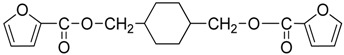
26.26	372	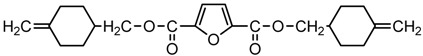
26.43	316	unidentified
27.90	390